# Poly[diaqua-μ_2_-isonicotinato-μ_2_-oxalato-terbium(III)]

**DOI:** 10.1107/S1600536808042682

**Published:** 2008-12-20

**Authors:** Zhan-Qiang Fang, Rong-Hua Zeng, Yan-Ting Li, Shuo Yang, Zhao-Feng Song

**Affiliations:** a School of Chemistry and the Environment, South China Normal University, Guangzhou 510006, People’s Republic of China

## Abstract

In the crystal structure of the title complex, [Tb(C_6_H_4_NO_2_)(C_2_O_4_)(H_2_O)_2_]_*n*_, the Tb^III^ ion is coordinated by two O atoms from two isonicotinate (inic) anions, four O atoms of two oxalate anions, and two water mol­ecules, displaying a distorted square-antiprismatic geometry. The Tb^III^ ion, the inic anion and the water mol­ecules occupy general positions. One of the two crystallographically independent oxalate anions is located on a center of inversion, whereas the second is located on the twofold rotation axis. The carboxyl­ate groups of the inic and oxalate anions link the terbium metal centres into layers. These layers are connected by O—H⋯O and N—H⋯O hydrogen bonding into a three-dimensional network.

## Related literature

For background, see: Eddaoudi *et al.* (2001 ▶); Rizk *et al.* (2005 ▶). An independent determination of this structure is reported in the preceeding paper, see: Song *et al.* (2009 ▶).
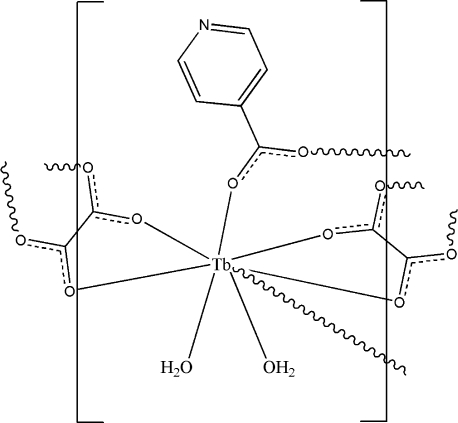


## Experimental

### 

#### Crystal data

[Tb(C_6_H_4_NO_2_)(C_2_O_4_)(H_2_O)_2_]
*M* *_r_* = 405.07
Monoclinic, 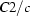

*a* = 17.7919 (18) Å
*b* = 9.9259 (10) Å
*c* = 12.9670 (13) Å
β = 112.4140 (10)°
*V* = 2117.0 (4) Å^3^
*Z* = 8
Mo *K*α radiation
μ = 6.72 mm^−1^
*T* = 296 (2) K
0.23 × 0.22 × 0.20 mm

#### Data collection

Bruker APEXII area-detector diffractometer
Absorption correction: multi-scan (*APEX2*; Bruker, 2004 ▶) *T*_min_ = 0.241, *T*_max_ = 0.272
5243 measured reflections
1907 independent reflections
1674 reflections with *I* > 2σ(*I*)
*R*_int_ = 0.031

#### Refinement

*R*[*F* ^2^ > 2σ(*F* ^2^)] = 0.025
*wR*(*F* ^2^) = 0.063
*S* = 1.01
1907 reflections
163 parameters
6 restraints
H-atom parameters constrained
Δρ_max_ = 1.40 e Å^−3^
Δρ_min_ = −1.31 e Å^−3^

### 

Data collection: *APEX2* (Bruker, 2004 ▶); cell refinement: *SAINT* (Bruker, 2004 ▶); data reduction: *SAINT*; program(s) used to solve structure: *SHELXS97* (Sheldrick, 2008 ▶); program(s) used to refine structure: *SHELXL97* (Sheldrick, 2008 ▶); molecular graphics: *PLATON* (Spek, 2003 ▶) and *SHELXTL* (Sheldrick, 2008 ▶); software used to prepare material for publication: *SHELXL97*.

## Supplementary Material

Crystal structure: contains datablocks I, global. DOI: 10.1107/S1600536808042682/nc2118sup1.cif

Structure factors: contains datablocks I. DOI: 10.1107/S1600536808042682/nc2118Isup2.hkl

Additional supplementary materials:  crystallographic information; 3D view; checkCIF report

## Figures and Tables

**Table 1:** Hydrogen-bond geometry (Å, °)

*D*—H⋯*A*	*D*—H	H⋯*A*	*D*⋯*A*	*D*—H⋯*A*
O1*W*—H2*W*⋯O3^i^	0.84	2.22	2.992 (5)	153
O2*W*—H4*W*⋯O1*W*^ii^	0.84	2.19	3.003 (5)	163
O1*W*—H1*W*⋯N1^iii^	0.84	1.83	2.661 (5)	167
O2*W*—H3*W*⋯O6^iv^	0.84	2.01	2.836 (5)	172

Symmetry codes: (i) 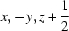; (ii) 
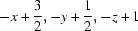
; (iii) 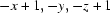; (iv) 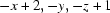.

**Fig. 1.**
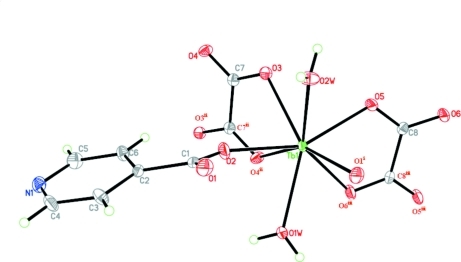
Part of the crystal structure of the title compound with labelling and displacement ellipsoids drawn at the 30% probabbility level. Symmetry codes: (i)1.5-x, 0.5-y, 1-z; (ii)1.5-x, -0.5-y, 1-z; (iii)2-x, y, 1.5-z;

**Fig. 2.**
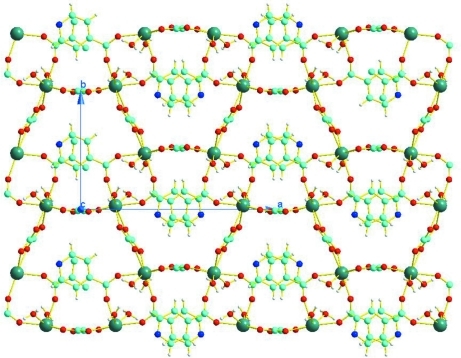
Crystal structure of the title compound with view along the c-axis.

## supplementary crystallographic information

### Comment

The design, synthesis, characterization and properties of coordination networks formed by functionalized organic molecules or anionas as bridges between metal centers are of great interest(Rizk *et al.*,  2005; Eddaoudi *et al.*, 2001). As a building block, isonicotinic acid and oxalic acid are excellent candidates for the construction of such compounds. In our ongoing investigations in this field the title compound was prepared and structurally characterized.

In the crystal structure of the title compound each Tb^III^ centre is coordinated by six oxygen atoms from two symmetry related inic anions, two crystallographically independent oxalate anions and two crystallographically independent water molecules within a distorted bicapped trigonal prismatic geometry (Fig. 1) . The Tb^III^ ions are linked by the inic and oxalate anions into layers, which are parallel to the b-c-plane (Fig. 2). Tb···Tb separations amount to 6.177 (4) and 5.047 (5) Å, respectively. These layers are connected via O—H···O and N—H···O hydrogen bonding between the water H atoms and the inic and one of the two crystallographically independent oxalate anions into three-dimensional network (Table 1).

### Experimental

A mixture of Tb_4_O_7_ (0.189 g; 0.25 mmol), isonicotinic acid (0.135 g; 1.5 mmol), oxalic acid(0.135 g; 1.5 mmol), water (10 mL) and HNO_3_ (0.385 mmol; 0.92g/ml) were stirred for 20 min and then sealed in a 20 mL Teflon-lined stainless-steel autoclave. The autoclave was heated to 433K for 3 days, and then cooled to room temperature at 5 K h^-1^. By this procedure colorless block-like crystals of the title compound were obtained.

### Refinement

The Water H atoms were located in difference Fourier maps, their bond lengths were set to ideal values of O–H = 0.84 and finally they were refined isotropic using a riding model with *U*_iso_(H) = 1.5 *U*_eq_(O). C-H H atoms were placed at calculated positions and were treated as riding on the parent C atoms with C—H = 0.93 Å, and with *U*_iso_(H) = 1.2 *U*_eq_(C).

### Crystal data

**Table:**

[Tb(C_6_H_4_NO_2_)(C_2_O_4_)(H_2_O)_2_]	*F*(000) = 1536
*M**_r_* = 405.07	*D*_x_ = 2.542 Mg m^−^^3^
Monoclinic, *C*2/*c*	Mo *K*α radiation, λ = 0.71073 Å
*a* = 17.7919 (18) Å	Cell parameters from 2410 reflections
*b* = 9.9259 (10) Å	θ = 2.4–27.7°
*c* = 12.9670 (13) Å	µ = 6.72 mm^−^^1^
β = 112.414 (1)°	*T* = 296 K
*V* = 2117.0 (4) Å^3^	Block, colourless
*Z* = 8	0.23 × 0.22 × 0.20 mm

### Data collection

**Table:**

Bruker APEXII area-detector diffractometer	1674 reflections with *I* > 2σ(*I*)
Radiation source: fine-focus sealed tube	*R*_int_ = 0.031
φ and ω scans	θ_max_ = 25.2°, θ_min_ = 2.4°
Absorption correction: multi-scan (*APEX2*; Bruker, 2004)	*h* = −21→20
*T*_min_ = 0.241, *T*_max_ = 0.272	*k* = −5→11
5243 measured reflections	*l* = −15→15
1907 independent reflections	

### Refinement

**Table:**

Refinement on *F*^2^	Primary atom site location: structure-invariant direct methods
Least-squares matrix: full	Secondary atom site location: difference Fourier map
*R*[*F*^2^ > 2σ(*F*^2^)] = 0.025	Hydrogen site location: inferred from neighbouring sites
*wR*(*F*^2^) = 0.063	H-atom parameters constrained
*S* = 1.01	*w* = 1/[σ^2^(*F*_o_^2^) + (0.0363*P*)^2^] where *P* = (*F*_o_^2^ + 2*F*_c_^2^)/3
1907 reflections	(Δ/σ)_max_ = 0.002
163 parameters	Δρ_max_ = 1.40 e Å^−^^3^
6 restraints	Δρ_min_ = −1.31 e Å^−^^3^

### Special details

**Table:**

Geometry. All esds (except the esd in the dihedral angle between two l.s. planes) are estimated using the full covariance matrix. The cell esds are taken into account individually in the estimation of esds in distances, angles and torsion angles; correlations between esds in cell parameters are only used when they are defined by crystal symmetry. An approximate (isotropic) treatment of cell esds is used for estimating esds involving l.s. planes.
Refinement. Refinement of F^2^ against ALL reflections. The weighted R-factor wR and goodness of fit S are based on F^2^, conventional R-factors R are based on F, with F set to zero for negative F^2^. The threshold expression of F^2^ > 2sigma(F^2^) is used only for calculating R-factors(gt) etc. and is not relevant to the choice of reflections for refinement. R-factors based on F^2^ are statistically about twice as large as those based on F, and R- factors based on ALL data will be even larger.

### Fractional atomic coordinates and isotropic or equivalent isotropic displacement parameters (Å^2^)

**Table:**

	*x*	*y*	*z*	*U*_iso_*/*U*_eq_	
Tb1	0.824056 (12)	0.03238 (2)	0.574780 (18)	0.01553 (10)	
O5	0.9618 (2)	−0.0185 (4)	0.6050 (3)	0.0289 (9)	
O2	0.6877 (2)	0.0558 (4)	0.4672 (3)	0.0326 (9)	
O3	0.8029 (2)	−0.1491 (3)	0.4403 (3)	0.0239 (8)	
C8	1.0149 (3)	−0.0110 (5)	0.7019 (4)	0.0192 (11)	
O6	1.0905 (2)	−0.0055 (4)	0.7291 (3)	0.0272 (9)	
C1	0.6278 (3)	0.1294 (5)	0.4198 (4)	0.0215 (11)	
C2	0.5442 (3)	0.0692 (5)	0.3902 (4)	0.0172 (10)	
C3	0.4756 (3)	0.1501 (6)	0.3627 (4)	0.0264 (12)	
H3	0.4800	0.2434	0.3618	0.032*	
C6	0.5336 (3)	−0.0690 (5)	0.3874 (4)	0.0241 (11)	
H6	0.5781	−0.1263	0.4050	0.029*	
C4	0.4010 (3)	0.0890 (6)	0.3366 (4)	0.0315 (13)	
H4	0.3556	0.1438	0.3206	0.038*	
C5	0.4563 (3)	−0.1211 (5)	0.3584 (5)	0.0314 (13)	
H5	0.4500	−0.2142	0.3564	0.038*	
N1	0.3903 (3)	−0.0438 (5)	0.3330 (4)	0.0309 (11)	
C7	0.7582 (3)	−0.2447 (5)	0.4457 (4)	0.0201 (11)	
O4	0.7261 (2)	−0.3320 (3)	0.3724 (3)	0.0252 (8)	
O1	0.6308 (2)	0.2507 (4)	0.3926 (3)	0.0334 (9)	
O1W	0.75857 (19)	0.1250 (3)	0.6941 (3)	0.0228 (8)	
H1W	0.7146	0.0877	0.6885	0.034*	
H2W	0.7794	0.1546	0.7598	0.034*	
O2W	0.8300 (2)	0.1188 (4)	0.4029 (3)	0.0312 (9)	
H3W	0.8554	0.0794	0.3691	0.047*	
H4W	0.8051	0.1834	0.3631	0.047*	

### Atomic displacement parameters (Å^2^)

**Table:**

	*U*^11^	*U*^22^	*U*^33^	*U*^12^	*U*^13^	*U*^23^
Tb1	0.01241 (14)	0.01564 (15)	0.01830 (15)	−0.00225 (9)	0.00558 (10)	−0.00106 (9)
O5	0.0153 (18)	0.048 (2)	0.023 (2)	0.0038 (17)	0.0067 (16)	−0.0063 (18)
O2	0.0144 (18)	0.052 (3)	0.028 (2)	0.0001 (18)	0.0045 (16)	0.001 (2)
O3	0.0271 (19)	0.0201 (18)	0.0287 (19)	−0.0097 (16)	0.0153 (16)	−0.0047 (16)
C8	0.017 (3)	0.018 (2)	0.019 (3)	0.003 (2)	0.003 (2)	0.000 (2)
O6	0.0132 (18)	0.040 (2)	0.027 (2)	0.0019 (16)	0.0068 (16)	−0.0024 (17)
C1	0.019 (3)	0.031 (3)	0.016 (2)	−0.006 (2)	0.009 (2)	−0.007 (2)
C2	0.017 (2)	0.021 (3)	0.014 (2)	−0.005 (2)	0.0058 (19)	0.001 (2)
C3	0.023 (3)	0.028 (3)	0.033 (3)	0.004 (2)	0.017 (2)	0.008 (2)
C6	0.019 (3)	0.022 (3)	0.031 (3)	−0.003 (2)	0.010 (2)	0.001 (2)
C4	0.019 (3)	0.049 (4)	0.030 (3)	0.007 (3)	0.014 (2)	0.008 (3)
C5	0.036 (3)	0.022 (3)	0.040 (3)	−0.013 (3)	0.018 (3)	−0.004 (3)
N1	0.023 (2)	0.044 (3)	0.026 (2)	−0.009 (2)	0.010 (2)	−0.001 (2)
C7	0.019 (2)	0.017 (3)	0.023 (3)	0.004 (2)	0.006 (2)	0.002 (2)
O4	0.033 (2)	0.0225 (18)	0.0218 (18)	−0.0081 (16)	0.0128 (16)	−0.0050 (16)
O1	0.038 (2)	0.027 (2)	0.041 (2)	−0.0189 (18)	0.0211 (19)	−0.0120 (19)
O1W	0.0180 (17)	0.027 (2)	0.0260 (18)	−0.0064 (15)	0.0116 (15)	−0.0071 (16)
O2W	0.049 (2)	0.024 (2)	0.029 (2)	0.0045 (18)	0.0241 (18)	0.0045 (17)

### Geometric parameters (Å, °)

**Table:**

Tb1—O1^i^	2.280 (3)	C2—C3	1.389 (7)
Tb1—O2	2.303 (3)	C3—C4	1.379 (7)
Tb1—O5	2.383 (3)	C3—H3	0.9300
Tb1—O4^ii^	2.385 (3)	C6—C5	1.380 (7)
Tb1—O2W	2.427 (3)	C6—H6	0.9300
Tb1—O3	2.435 (3)	C4—N1	1.330 (8)
Tb1—O6^iii^	2.443 (4)	C4—H4	0.9300
Tb1—O1W	2.443 (3)	C5—N1	1.335 (7)
O5—C8	1.254 (6)	C5—H5	0.9300
O2—C1	1.244 (6)	C7—O4	1.251 (6)
O3—C7	1.259 (6)	C7—C7^ii^	1.545 (10)
C8—O6	1.256 (6)	O4—Tb1^ii^	2.385 (3)
C8—C8^iii^	1.529 (10)	O1—Tb1^i^	2.280 (3)
O6—Tb1^iii^	2.443 (4)	O1W—H1W	0.8429
C1—O1	1.261 (6)	O1W—H2W	0.8420
C1—C2	1.511 (6)	O2W—H3W	0.8367
C2—C6	1.383 (7)	O2W—H4W	0.8365
			
O1^i^—Tb1—O2	103.43 (14)	O6—C8—C8^iii^	116.0 (5)
O1^i^—Tb1—O5	84.39 (13)	C8—O6—Tb1^iii^	118.3 (3)
O2—Tb1—O5	154.22 (14)	O2—C1—O1	125.4 (5)
O1^i^—Tb1—O4^ii^	151.43 (12)	O2—C1—C2	117.9 (5)
O2—Tb1—O4^ii^	80.50 (13)	O1—C1—C2	116.7 (5)
O5—Tb1—O4^ii^	104.46 (13)	C6—C2—C3	118.0 (4)
O1^i^—Tb1—O2W	72.60 (12)	C6—C2—C1	120.7 (4)
O2—Tb1—O2W	79.21 (13)	C3—C2—C1	121.3 (5)
O5—Tb1—O2W	79.88 (13)	C4—C3—C2	118.6 (5)
O4^ii^—Tb1—O2W	135.25 (12)	C4—C3—H3	120.7
O1^i^—Tb1—O3	141.11 (12)	C2—C3—H3	120.7
O2—Tb1—O3	78.56 (13)	C5—C6—C2	119.4 (5)
O5—Tb1—O3	80.16 (12)	C5—C6—H6	120.3
O4^ii^—Tb1—O3	67.43 (11)	C2—C6—H6	120.3
O2W—Tb1—O3	69.67 (11)	N1—C4—C3	123.8 (5)
O1^i^—Tb1—O6^iii^	85.24 (13)	N1—C4—H4	118.1
O2—Tb1—O6^iii^	137.67 (13)	C3—C4—H4	118.1
O5—Tb1—O6^iii^	66.65 (12)	N1—C5—C6	122.9 (5)
O4^ii^—Tb1—O6^iii^	74.06 (12)	N1—C5—H5	118.6
O2W—Tb1—O6^iii^	141.48 (12)	C6—C5—H5	118.6
O3—Tb1—O6^iii^	119.77 (12)	C4—N1—C5	117.4 (4)
O1^i^—Tb1—O1W	75.34 (12)	O4—C7—O3	126.5 (5)
O2—Tb1—O1W	72.48 (13)	O4—C7—C7^ii^	117.1 (5)
O5—Tb1—O1W	133.16 (12)	O3—C7—C7^ii^	116.4 (5)
O4^ii^—Tb1—O1W	79.12 (11)	C7—O4—Tb1^ii^	118.1 (3)
O2W—Tb1—O1W	130.27 (12)	C1—O1—Tb1^i^	154.0 (4)
O3—Tb1—O1W	138.71 (11)	Tb1—O1W—H1W	115.5
O6^iii^—Tb1—O1W	69.91 (12)	Tb1—O1W—H2W	129.8
C8—O5—Tb1	119.1 (3)	H1W—O1W—H2W	106.0
C1—O2—Tb1	149.9 (4)	Tb1—O2W—H3W	122.5
C7—O3—Tb1	116.5 (3)	Tb1—O2W—H4W	129.8
O5—C8—O6	127.0 (5)	H3W—O2W—H4W	107.4
O5—C8—C8^iii^	117.0 (5)		

Symmetry codes: (i) −*x*+3/2, −*y*+1/2, −*z*+1; (ii) −*x*+3/2, −*y*−1/2, −*z*+1; (iii) −*x*+2, *y*, −*z*+3/2.

### Hydrogen-bond geometry (Å, °)

**Table:**

*D*—H···*A*	*D*—H	H···*A*	*D*···*A*	*D*—H···*A*
O1W—H2W···O3^iv^	0.84	2.22	2.992 (5)	153
O2W—H4W···O1W^i^	0.84	2.19	3.003 (5)	163
O1W—H1W···N1^v^	0.84	1.83	2.661 (5)	167
O2W—H3W···O6^vi^	0.84	2.01	2.836 (5)	172

Symmetry codes: (iv) *x*, −*y*, *z*+1/2; (i) −*x*+3/2, −*y*+1/2, −*z*+1; (v) −*x*+1, −*y*, −*z*+1; (vi) −*x*+2, −*y*, −*z*+1.
